# Portraits of a pressure sensor

**DOI:** 10.7554/eLife.34396

**Published:** 2018-01-29

**Authors:** Alexander T Chesler, Marcin Szczot

**Affiliations:** National Center of Complementary and Integrative HealthNational Institutes of HealthBethesdaUnited States

**Keywords:** piezo ion channels, mechanosensitivity, cryoEM, somatosensation, vascular system, Human, Mouse

## Abstract

Near atomic-resolution structures have provided insights into the mechanisms by which the Piezo1 ion channel senses and responds to mechanical stimuli.

**Related research article** Guo YR, MacKinnon R. 2017. Structure-based membrane dome mechanism for Piezo mechanosensitivity. *eLife* **6**:e33660. doi: 10.7554/eLife.33660

From our lungs and bladder to our bones and blood, many organs and structures in the body have to be sensitive to mechanical pressure in order to detect and respond to both internal and external signals. At the level of the cell, changes in mechanical pressure are detected by ion channels – structures in the membrane that let ions move into or out of cells. In mechanosensitive ion channels, pressure on the membrane triggers the opening or the closing of the channels, converting changes in membrane tension into electrochemical signals.

Piezo1 and Piezo2 are two mechanosensitive ion channels that are involved in processes as diverse as perceiving touch or regulating the volume of red blood cells in mammals (Murthy et al., 2017). Moreover, defects in Piezo1 or Piezo2 can lead to a variety of diseases, including a number of blood disorders (Gallagher, 2013) and problems with proprioception (Chesler et al., 2016).

With over 2,000 amino acids that span the cell membrane dozens of times, both Piezo proteins are unusually large molecules. Further, they do not share any known structural similarity with other mammalian proteins. These particularities have made it difficult to understand their structure and to study how the channels sense and respond to mechanical stimulation.

Now, in eLife and Nature, three independent groups – Yusong Guo and Rod Mackinnon of Rockefeller University (Guo and MacKinnon, 2017), Ardem Patapoputian, Andrew Ward and co-workers at the Scripps Research Institute (Saotome et al., 2017), and Xueming Li, Bailong Xiao and co-workers at the Tsinghua-Peking Joint Center for Life Sciences (Zhao et al., 2018) – report that they have used cryo-electron microscopy to determine the structure of the mammalian Piezo1 channel in the closed state with near atomic resolution.

Piezo ion channels are trimers and are shaped like a propeller with three blades organized around a central pore (Figure 1A; Ge et al., 2015). The study by Guo and Mackinnon finds that the Piezo1 molecules bend the local lipid environment to form 'dome-like' structures. The implication is that, when the channel is closed, the three blades curve out of the plane of the pore domain. This physically bows the membrane, creating the dome. They propose that as the tension on the membrane increases, the dome flattens and the blades straighten; this could supply the energy needed to open the channel (Figure 1C). In support of this model, Zhao et al. report that the propellers can indeed adopt several distinct conformations. The central pore also appears to be well insulated from the parts of the ion channel that can detect mechanical forces. This means that many of the properties of the channel – including its sensitivity and selectivity – would be preserved under a range of membrane tensions.

**Figure 1.**
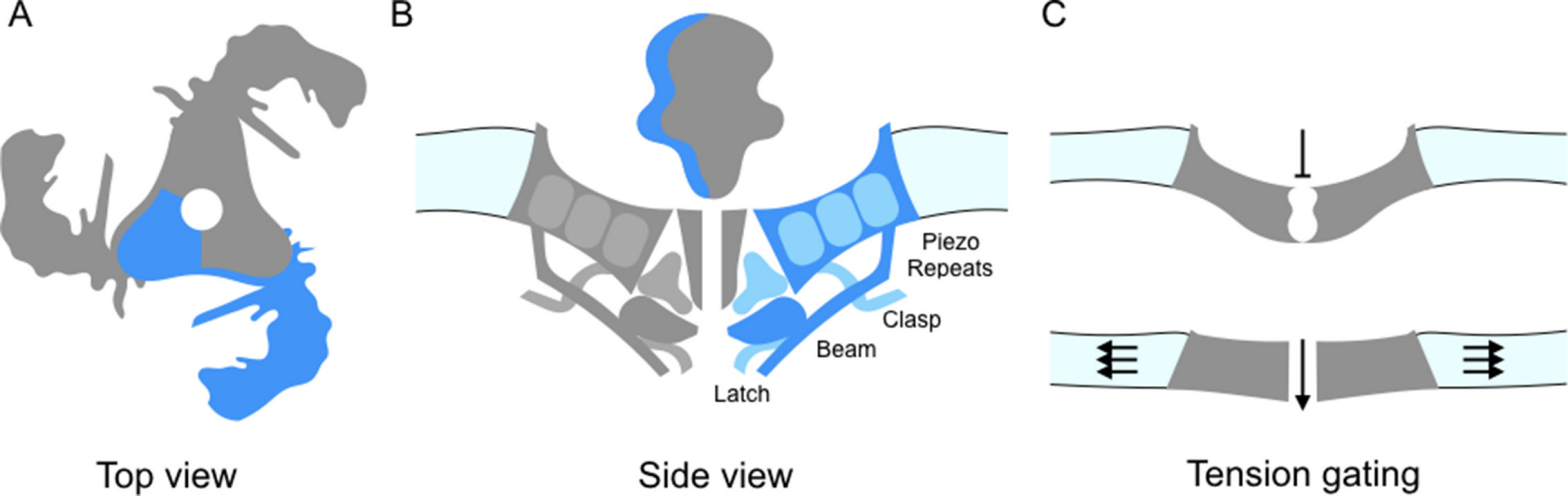
Details of the Piezo1 ion channel. (**A**) The schematic structure of Piezo1 viewed from above, showing the three 'propeller blades' surrounding a central pore. A single propeller blade is highlighted in blue. (**B**) A side view of the structure of Piezo1 as revealed by cryo-electron microscopy, with a single propeller blade highlighted in dark and medium blue: the interior of the cell is at the bottom of the figure. Each propeller blade contains at least six piezo-repeats, but only the three nearest to the central pore are shown (medium blue). Each propeller blade also includes a 'beam' domain (dark blue) that is parallel to the cell membrane, and a structure called the 'latch' (dark blue) that is in contact with the intracellular ends of the inner helices (dark grey) that form the central pore. Each propeller also contains a 'clasp' domain (medium blue): this domain interacts further from the pore, but its structure has not been determined yet. (**C**) When the cell is not submitted to pressure, Piezo1 bends the membrane to make a dome-like structure pointing inside the cell, and the channel is closed. When the membrane is stretched the complex flattens out, opening the channel.

In turn, the work by all three groups allow a more precise description of the structure of Piezo1, in particular of its core architecture. Each propeller blade is made up of 6-9 similar units (which Saotome et al. call 'piezo-repeats'), with each unit being composed of four helical bundles (Figure 1B). The intracellular end of the piezo-repeats connects to helices parallel to the membrane, which is an ideal position from which to influence and respond to membrane tension. On the inner part of the blade, closer to the pore, the 'beam' domain (previously described by Ge et al., 2015) runs close to the piezo-repeats near to the pore and looks suspiciously like a support brace or lever. Zhao et al. demonstrate that mutations in the beam lead to a reduction in the mechanical sensitivity. The beam associates with two newly resolved regions (called 'latch' and 'clasp') which may act as fulcrums or hinges.

Remarkably, the linker that connects the beam and the latch is created by a large intracellular loop that seems to play a key role in opening the pore. In Piezo2, an inherited mutation in this loop leads to a loss of function (Chesler et al., 2016), while alternative variants of this area alter key biophysical properties of the channels such as their rate of inactivation (Szczot et al., 2017). It is possible that the deformations in the membrane are transmitted from the outer part of the blades to the center of the channel through this complex assemblage of protein domains, including the beam.

Moving forward, it remains to be determined whether Piezo1 and Piezo2 adopt similar conformations in a native cell environment (as the experiments by Guo and Mackinnon were performed in reconstituted systems). Yet, the structures revealed by both groups certainly will be invaluable tools to guide future structure/function and mutagenesis experiments. For one, they could help with understanding better how the channels are regulated. Inactivation of Piezos is fast compared to other sensory ion channels, on par with synaptic receptors like AMPA or GABA_A_. Inactivation may be determined by the extracellular domain (Wu et al., 2017) but intracellular (Szczot et al., 2017) and pore (Saotome et al., 2017) residues can also clearly affect it.

To date, only one molecule has been found to chemically regulate the opening of these channels: Yoda-1, which artificially activates Piezo1 (Syeda et al., 2015). Determining its site of action should prove particularly informative. A better understanding of the structure of Piezo1 will hopefully help guide screens to find new chemical agonists, antagonists and/or modulators. With so much still unknown about how these molecules function, identifying new mutations and pharmacological tools that slow down or remove inactivation would greatly facilitate determining other channel conformations, particular the open state, as well as testing models of mechanosensing.

## References

[bib1] Chesler AT, Szczot M, Bharucha-Goebel D, Čeko M, Donkervoort S, Laubacher C, Hayes LH, Alter K, Zampieri C, Stanley C, Innes AM, Mah JK, Grosmann CM, Bradley N, Nguyen D, Foley AR, Le Pichon CE, Bönnemann CG (2016). The role of PIEZO2 in human mechanosensation. The New England Journal of Medicine.

[bib2] Gallagher PG (2013). Disorders of red cell volume regulation. Current Opinion in Hematology.

[bib3] Ge J, Li W, Zhao Q, Li N, Chen M, Zhi P, Li R, Gao N, Xiao B, Yang M (2015). Architecture of the mammalian mechanosensitive Piezo1 channel. Nature.

[bib4] Guo YR, MacKinnon R (2017). Structure-based membrane dome mechanism for Piezo mechanosensitivity. eLife.

[bib5] Murthy SE, Dubin AE, Patapoutian A (2017). Piezos thrive under pressure: mechanically activated ion channels in health and disease. Nature Reviews Molecular Cell Biology.

[bib6] Saotome K, Murthy SE, Kefauver JM, Whitwam T, Patapoutian A, Ward AB (2017). Structure of the mechanically activated ion channel Piezo1. Nature.

[bib7] Syeda R, Xu J, Dubin AE, Coste B, Mathur J, Huynh T, Matzen J, Lao J, Tully DC, Engels IH, Petrassi HM, Schumacher AM, Montal M, Bandell M, Patapoutian A (2015). Chemical activation of the mechanotransduction channel Piezo1. eLife.

[bib8] Szczot M, Pogorzala LA, Solinski HJ, Young L, Yee P, Le Pichon CE, Chesler AT, Hoon MA (2017). Cell-Type-Specific splicing of Piezo2 regulates mechanotransduction. Cell Reports.

[bib9] Wu J, Young M, Lewis AH, Martfeld AN, Kalmeta B, Grandl J (2017). Inactivation of mechanically activated Piezo1 ion channels is determined by the C-terminal extracellular domain and the inner pore helix. Cell Reports.

[bib10] Zhao Q, Zhou H, Chi S, Wang Y, Wang J, Geng J, Wu K, Liu W, Zhang T, Dong M, Wang J, Li X, Xiao B (2018). Structure and mechanogating mechanism of the Piezo1 channel. Nature.

